# A survey of multilingual large language models

**DOI:** 10.1016/j.patter.2024.101118

**Published:** 2025-01-10

**Authors:** Libo Qin, Qiguang Chen, Yuhang Zhou, Zhi Chen, Yinghui Li, Lizi Liao, Min Li, Wanxiang Che, Philip S. Yu

**Affiliations:** 1 School of Computer Science and Engineering, Central South University, Changsha 410083, China; 2 Research Center for Social Computing and Information Retrieval, Harbin Institute of Technology, Harbin 150001, China; 3 ByteDance, Inc., Shanghai 200082, China; 4 Tsinghua Shenzhen International Graduate School, Tsinghua University, Shenzhen 518055, China; 5 School of Computing and Information Systems, Singapore Management University, Singapore 188065, Singapore; 6 Department of Computer Science, University of Illinois at Chicago, Chicago, IL 60637, USA

**Keywords:** multilingual large language model, large language model, cross-lingual transfer, multilingual alignment, parameter-tuning alignment, parameter-frozen alignment

## Abstract

Multilingual large language models (MLLMs) leverage advanced large language models to process and respond to queries across multiple languages, achieving significant success in polyglot tasks. Despite these breakthroughs, a comprehensive survey summarizing existing approaches and recent developments remains absent. To this end, this paper presents a unified and thorough review of the field, highlighting recent progress and emerging trends in MLLM research. The contributions of this paper are as follows. (1) Extensive survey: to our knowledge, this is the pioneering thorough review of multilingual alignment in MLLMs. (2) Unified taxonomy: we provide a unified framework to summarize the current progress in MLLMs. (3) Emerging frontiers: key emerging frontiers are identified, alongside a discussion of associated challenges. (4) Abundant resources: we collect abundant open-source resources, including relevant papers, data corpora, and leaderboards. We hope our work can provide the community quick access and spur breakthrough research in MLLMs.

## The bigger picture

The rapid advancement of large language models (LLMs) has significantly transformed natural language processing (NLP), enabling machines to understand and generate human-like text. However, most LLMs are predominantly English centric, limiting their applicability in our linguistically diverse world. With over 7,000 languages spoken globally, there is a pressing need for models that can comprehend and generate text across multiple languages. Multilingual large language models (MLLMs) address this gap by processing and producing content in various languages, thereby enhancing global communication and accessibility. This survey provides a comprehensive overview of MLLMs, introducing a systematic taxonomy based on alignment strategies to deepen understanding in this field. By highlighting emerging trends and challenges, this survey aims to guide future research and development, fostering the creation of more inclusive and effective language models that cater to the diverse linguistic landscape of our world.

## Teaser

This survey explores the growing field of multilingual large language models (MLLMs), addressing the challenges of linguistic diversity in natural language processing. By introducing a systematic taxonomy based on alignment strategies, the authors provide a structured understanding of MLLMs' capabilities. The paper reviews current advancements, identifies emerging trends, and outlines challenges in building inclusive language models. This comprehensive overview offers critical insights to guide future research and development, promoting more equitable and effective multilingual communication in an increasingly interconnected world.

## Introduction

In recent years, remarkable progress has been witnessed in large language models (LLMs),^1^^,^^2^^,^^3^^,^^4^ which have achieved excellent performance in various natural language processing tasks.^5^^,^^6^^,^^7^ In addition, LLMs raise surprising emergent capabilities, including in-context learning,^8^^,^^9^ chain-of-thought reasoning,^10^^,^^11^^,^^12^ and even planning.^13^^,^^14^ Nevertheless, the majority of LLMs are English centric, primarily focusing on English tasks,^15^^,^^16^ which renders them relatively weak in multilingual settings, especially in low-resource scenarios.

**Figure 1:**
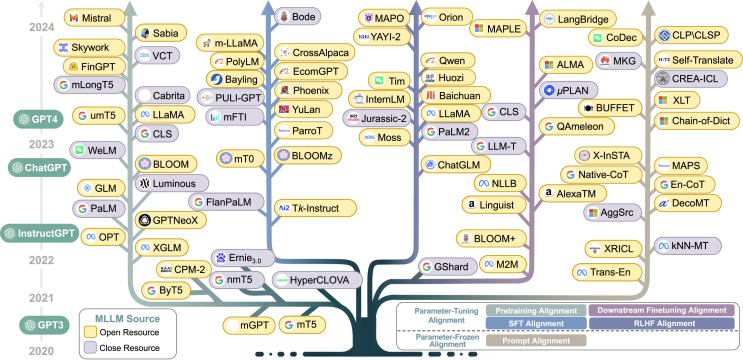
Evolution of selected MLLMs over the past 5 years, where colored branches indicate different alignment stages For models with multiple alignment stages, the final stage is represented.

Actually, there are over 7,000 languages in the world. With the acceleration of globalization, the success of LLMs should be leveraged to serve diverse countries and languages. To this end, as shown in Figure 1, multilingual large language models (MLLMs) possess the advantage of comprehensively handling multiple languages, gaining increasing attention. Specifically, existing MLLMs can be broadly divided into two groups based on different stages. The first series of works^17^^,^^18^^,^^19^^,^^20^ leverages multilingual data to tune parameters and boost the overall multilingual performance. The second series of works^11^^,^^12^^,^^21^ also adapts advanced prompting strategies to unlock the deeper multilingual potential of MLLMs during the parameter-frozen inference stage.

**Figure 2:**
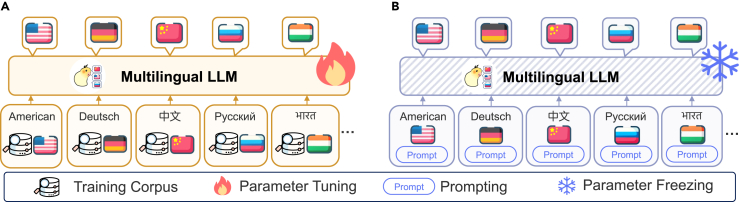
Parameter-tuning alignment vs. parameter-frozen alignment (A) Parameter-tuning alignment requires the model to fine-tune the MLLM parameters for cross-lingual alignment. (B) Parameter-frozen alignment directly uses prompts for alignment without parameter tuning.

While remarkable success has been achieved in MLLMs, there remains a lack of comprehensive review and analysis of recent efforts in the literature, which hinders the development of MLLMs. To bridge this gap, we attempt to conduct an extensive and detailed analysis of MLLMs. Specifically, we first introduce the widely used data resources and evaluation techniques. Furthermore, due to the key challenge of alignment across languages, we introduce a novel taxonomy according to alignment strategies, aiming to provide a unified perspective in the literature. This taxonomy primarily includes parameter-tuning alignment (PTA) and parameter-frozen alignment (PFA; as shown in Figure 2). In particular, PTA involves fine-tuning model parameters to enhance alignment between English and target languages during pretraining, supervised fine-tuning (SFT), reinforcement learning from human feedback (RLHF), and downstream fine-tuning. On the other hand, PFA refers to alignment achieved by prompting across languages, without requiring adjustments to model parameters. Finally, we highlight some potential frontier areas and the corresponding challenges, especially MLLMs for low-resource languages, hoping to inspire follow-up research.

The main contributions of this work can be summarized as follows. (1) Extensive survey: to the best of our knowledge, we present a comprehensive survey in the MLLM literature based on multilingual alignment. (2) Unified taxonomy: we introduce a unified taxonomy categorizing MLLMs into two alignment types, parameter frozen and parameter tuning, providing a systematic perspective to understand the MLLM literature. (3) Emerging frontiers: we discuss emerging frontiers and highlight their challenges as well as their opportunities, aiming to pave the way for future research developments. (4) Abundant resources: we attempt to organize MLLM resources, including open-source software, diverse corpora, and a curated list of relevant publications.

We hope this work can serve as a valuable resource for researchers and inspire further breakthroughs in future research.

## Related work

Multilingual and cross-lingual natural language processing (NLP) has emerged as a vibrant research area.^22^^,^^23^^,^^24^ Recent surveys have shed light on multilingual models and cross-lingual transfer, examining language technologies in diverse linguistic and cultural contexts. Previously, Doddapaneni et al.^25^ demonstrated that pretrained language models (PLMs) enhance performance in both familiar and unfamiliar languages across various tasks. Similarly, Philippy et al.^26^ analyzed the factors affecting zero-shot cross-lingual transfer, offering an in-depth discussion. Additionally, Doğruöz et al.^27^ and Winata et al.^28^ explored the linguistic and social dynamics of code switching, highlighting its significance in multilingual NLP. The rising demand for global multilingual systems has spurred numerous downstream tasks. For instance, Dabre et al.^24^ examined PLMs in machine translation, while Deng et al.^29^ focused on their role in information extraction. Panchendrarajan and Zubiaga^30^ reviewed methods for identifying fact claims in multilingual and cross-lingual settings. As PLMs are deployed in real-world applications, concerns regarding safety, fairness, and bias have grown. Hershcovich et al.^31^ emphasized the cultural sensitivities crucial for effective cross-lingual NLP, while Jiang and Zubiaga^32^ discussed offensive language management and dataset challenges. Navigli et al.^33^ and Ramesh et al.^34^ highlighted the risks of bias, particularly in non-English languages, stressing the need for fairness in multilingual models. Lastly, Yadav and Sitaram^35^ expanded their reviews of multilingual PLMs to include multi-modal scenarios. In summary, existing surveys provide comprehensive insights into the technical advancements and challenges of multilingual PLMs, calling for a deeper understanding of these models across diverse cultural and linguistic environments.

With the emergence of LLMs such as GPT-3^1^ and GPT-4,^36^ various surveys have examined the architecture, capabilities, and limitations of these models, with a particular emphasis on multilingual performance and their alignment with human-like understanding.^4^^,^^37^^,^^38^^,^^39^ Nonetheless, there is a notable lack of comprehensive surveys specifically focused on MLLMs. To address this gap, we undertake a systematic analysis of MLLMs within the contemporary landscape of LLMs.

## Preliminary definitions

In this section, we formally describe the definitions of monolingual LLMs and MLLMs.

### Monolingual LLMs

**Figure 3:**
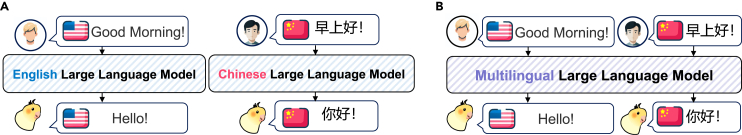
Monolingual large language model vs. multilingual LLM (A) Monolingual large language model (LLM) can only process one language at a time. (B) A multilingual LLM is capable of handling and producing content in various languages simultaneously, like English and Chinese.

A monolingual LLM can process only one language at a time. For example, as illustrated in Figure 3A, an English LLM and a Chinese LLM can handle English and Chinese languages separately. Formally, considering a set of languages $L={\left\{L_{i}\right\}}_{i=0}^{\left|L\right|}$, given an input utterance $X_{i}\in L_{i}$ in languages $L_{i}$, the process of a monolingual LLM ($M_{mono}$) generating the output $Y_{i}$ can be defined as

(Equation 1) $$Y_{i}=\left\{ \begin{array}{cc} M_{mono}\left(X_{i},L_{i}\right), & mono=L_{i};\\ Unexpect, & mono\neq L_{i}, \end{array}\right.$$

where *Unexpect* indicates that the LLM generates an unexpected output in an unintended language; *mono* denotes the single language that the LLM can correctly process.

### MLLMs

As shown in Figure 3B, unlike a monolingual LLM, an MLLM is capable of handling and producing content in various languages simultaneously, such as English and Chinese. Formally, for an MLLM $M_{multi}$, where multi⊆L and |multi|≥2, the multilingual response of the MLLM is given by

(Equation 2) $$Y=M_{multi}\left(X\right)\text{,}$$

where X and Y belong to multiple languages in *multi*.

## Resources for training

In this section, we describe the widely used data resources in pretraining, SFT, and RLHF stages in MLLMs.^4^

### Multilingual pretraining data

As shown in Table 1, the widely used multilingual corpora for pretraining in MLLMs can be divided into three categories: (1) manual creation: high-quality pretraining corpora obtained through manual creation and proofreading, including the Bible Corpus^40^ and MultiUN.^41^ (2) Web crawling: this involves crawling extensive multilingual data from the internet, which include OSCAR,^42^ CC-100,^43^ mC4,^17^ and Redpajama-v.2.^44^ Relatively speaking, the data quality obtained through extensive crawling is often poor; however, the sheer volume of data compensates for this by providing substantial world knowledge and long-tail knowledge. Another category of data is extracted from Wikipedia to enhance the knowledge embedded in MLLMs. Common datasets include Wikipedia, WikiMatrix,^45^ and WikiExpl.^46^ Since Wiki data are authored by humans, they feature high quality and sufficient knowledge density, making them a crucial resource for injecting knowledge into MLLMs. (3) Benchmark adaptation: this refers to re-cleaning or integrating existing benchmarks to enhance data quality, which includes datasets such as OPUS-100,^47^ Culturax,^48^ OPUS,^49^ WMT,^50^ and ROOTS.^51^ The pretraining data produced by this method are of higher quality than web-crawled data. However, this also results in such data being relatively scarce and lacking diversity.

**Table 1:** Pretraining data resources

Dataset	Storage size (B)	Token size (T)	Language size	Source	Latest update time
**Manual**					

Bible Corpus^40^	5.2 G	–	833	–	May 2014
MultiUN^41^	–	0.3 B	7	–	December 2014
IIT Bombay^52^	–	0.04 B	2	–	December 2021

**Web crawling**					

CC-100^43^	–	208 B	116	CommonCrawl	October 2022
mC4^17^	38.5 T	6.3 T	101	CommonCrawl	October 2022
Redpajama-v2^44^	30.4 T	–	5	CommonCrawl	December 2023
OSCAR^42^	6.3 T	800 B	166	CommonCrawl	January 2023
Oromo^53^	0.939 G	0.1 B	11	CommonCrawl	February 2022
Wu Dao 2.0	–	24 B	2	CommonCrawl	October 2023
MADLAD-400^54^	–	3 T	419	CommonCrawl	August 2022
HPLT-Full^55^	230.7 T	5.6 T	75	CommonCrawl	October 2022
HPLT-En-Center^55^	–	1.4 B	18	CommonCrawl	October 2023
Europarl^56^	1.5 G	0.6 B	21	–	May 2012
JW300^57^	–	1.5 B	343	–	July 2019
Glot500^58^	600 G	–	511	–	May 2023
Wikipedia^59^	–	24 B	300	Wikipedia	–
WikiMatrix^45^	65 G	–	85	Wikipedia	April 2021
OPUS-100^47^	2.6 G	–	100	OPUS	July 2020
AfricanNews^60^	12.3 G	–	16	mC4	September 2023
Taxi1500^61^	–	–	1500	Bible Corpus	May 2023
CulturaX^48^	27 T	6.3 T	167	mC4, OSCAR	January 2024
MMedC^62^	–	25.5 B	6	medical website	–

**Benchmark adaptation**					

ROOTS^51^	1.6 T	–	46	Huggingface	June 2022
OPUS^49^	–	40 B	1,304	–	December 2021
CCMT^63^	–	–	6	–	–
WMT^50^	–	–	32	–	–
IWSLT^64^	4.2 G	–	10	–	–

The term “source” refers to the origin datasets from which the pretraining data are derived. The storage size is measured in bytes (B); where relevant, "G" represents the billion scale and "T" represents the trillion scale. The token size is measured in the number of tokens (T); where relevant, "B" represents the billion scale and "T" represents the trillion scale.

### Multilingual SFT data

Similarly, as shown in Table 2, we categorize the existing multilingual SFT data into four classes: (1) manual creation: this involves acquiring SFT corpora through manual creation and proofreading, which includes Sup-NatInst,^65^ OpenAssist,^66^ and COIG-PC_lite_. This method ensures the highest quality, but it is costlier and produces a smaller volume of labeled data. (2) Machine translation: this method translates existing monolingual datasets into multilingual instruction datasets, which comprise xP3-MT,^20^ MGSM8K_Instruct_,^67^ CrossAlpaca,^68^^,^^69^ MultilingualSIFT,^70^ and Bactrain-X.^71^ This approach generates extremely large quantities of data with moderate quality. Its advantage lies in rapidly producing a substantial amount of non-English SFT data. However, it may fail to account for the cultural background of specific languages, leading to implicit biases. (3) Benchmark adaptation: this method transforms from existing benchmarks into an instruction format. Widely used datasets include xP3,^20^ PolyglotPrompt,^72^ and BUFFET.^73^ The data quality of this approach is high, but the diversity of tasks and instructions is limited. (4) MLLM-aided generation: such a strategy means that the data are automatically synthesized by MLLMs, containing Vicuna,^74^ OverMiss,^75^ ShareGPT, BELLE,^76^ MultiAlpaca,^77^ Guanaco,^78^ and Alpaca-4.^79^ Data generated from advanced MLLMs may surpass translation quality in high-resource languages. However, it is concerning that data quality may degrade in low-resource languages.

**Table 2:** Supervised fine-tuning data resource

Dataset	Sample size	Multilingual instruction	Language size	Task size
**Manual**				

Sup-NatInst^65^	–	–	55	1,616
OpenAssist^66^	–	–	35	–
EcomInstruct^80^	2.5 M	yes	2	12
COIG-PC-lite	650 K	no	2	3,250
Aya Dataset^81^	204 K	no	65	–

**Benchmark adaption**				

xP3^20^	80 M	no	46	71
BUFFET^73^	–	–	54	15
PolyglotPrompt^72^	–	no	49	6

**Translation**				

xP3-MT^20^	80 M	yes	46	71
xP3x^82^	168 M	no	101	56
Aya Collection^81^	513 M	no	114	44
MultilingualSIFT^70^	–	yes	11	–
Bactrian-X^71^	–	yes	52	–
MuIT^83^	–	yes	6	–
CrossAlpaca^68^	–	–	6	–
MGSM8KInstruct^67^	73.6 K	yes	6	10
XCoT^84^	7.4 K	yes	10	2

**MLLM aided**				

ShareGPT	–	–	–	–
Vicuna^74^	–	–	–	–
OverMiss^75^	54 K	–	3	1 (translation)
MultiAlpaca^77^	133 K	–	11	–
Guanaco^78^	535 K	–	5	–
Alpaca-4^79^	52 K	–	2	–

The term “multilingual instruction” denotes the presence of instructions in multiple languages to form the specific data input.

### Multilingual RLHF data

Furthermore, some works leverage multilingual RLHF data to improve alignment. Specifically, Lai et al.^85^ leverage multilingual ranking data to train a reward model using RLHF. Similarly, Zeng et al.^86^ introduce the TIM dataset to train a more effective reward model in multilingual contexts. This type of data is often labeled with preferences for translation tasks and is of high quality, though there remains room for improvement in task diversity.

## Multilingual performance evaluation

To facilitate the comparison of LLMs, extensive efforts have been dedicated to exploring enhanced evaluation methods for multilingual scenarios. This discussion elaborates on MLLM evaluation, covering both (1) evaluation metrics and (2) evaluation benchmarks.

### Evaluation metrics

#### Traditional automatic metric

Using traditional automatic metrics means that we assess the predicted output using decoding probabilities or PLM logits.^87^^,^^88^ In general, researchers utilize BLEU,^89^ BLEURT,^90^ chrF++,^91^ and COMET^92^ for translation evaluation and ROUGE^93^ for summary evaluation. Further, Guerreiro et al.^94^ propose xCOMET for improved translation evaluation through fine-grained error detection. To assess the general quality of the generated text, the commonly employed approach is to utilize multilingual BERTScore^95^ as an evaluation metric. Qin et al.^12^ extend Roscoe^96^ to multi-language for quality assessment of multilingual CoT. Furthermore, Hlavnova and Ruder^97^ develop a comprehensive and robust multilingual checklist system to thoroughly assess the MLLMs’ performance.

#### MLLM-based automatic metric

This approach employs robust MLLMs to score or compare generated outputs for evaluation purposes.^19^^,^^71^^,^^98^ Specifically, Zheng et al.^99^ introduce LLM-as-a-judge, where GPT-4 is prompted to assess the performance of other LLMs by comparing its output to the predicted one. However, this method remains unreliable in multilingual settings.^3^^,^^26^^,^^100^^,^^101^ Moreover, caution should be exercised particularly in languages where the MLLM is known to perform poorly.^102^ Furthermore, Kim et al.^103^ and Muller et al.^104^ conduct an attribution evaluation to thoroughly assess the robustness of the model.

#### Human evaluation

Human evaluation involves manually assessing MLLMs through detailed evaluations.^19^^,^^71^^,^^105^^,^^106^ Specifically, Lyu et al.^107^ initially explore the multilingual challenges of ChatGPT through manually annotated cases. Furthermore, Hu et al.^108^ introduce a new multilingual platform to facilitate more convenient manual assessments.

### Evaluation benchmarks

Current MLLMs tend to focus more on the alignment performance of non-English languages. Based on different perspectives of alignment, we categorize this into two areas: (1) natural language understanding and (2) natural language generation.

#### Natural language understanding

*Linguistics analysis*. For multilingual models, the fundamental requirement is understanding the linguistic differences across languages.^109^ The most common multilingual linguistic assessments include part-of-speech (POS) tagging,^110^^,^^111^ grammar analysis,^112^^,^^113^^,^^114^ and morphology.^115^ Additionally, Zhang et al.^116^ and Song et al.^117^ provide a comprehensive evaluation of the linguistic acceptability of MLLMs across languages.

*Semantic understanding*. Compared with linguistics analysis, researchers are more focused on the ability to analyze and understand the specific semantics of multiple languages.^30^^,^^100^^,^^118^^,^^119^^,^^120^ The most fundamental aspect of multilingual NLP is performing local semantic understanding, with the most typical task being information extraction,^121^ including datasets such as masakhaNER,^122^ MASSIVE,^123^ MultiCoNER,^124^^,^^125^ WikiAnn,^126^ and SMiLER.^127^ The second level involves a semantic understanding of complete sentences, which includes tasks like XNLI,^128^ Paws-X,^129^ MixATIS++,^130^ MTOP,^131^ MultiNLU,^132^ and PRESTO.^133^ Finally, semantic understanding can be further extended to paragraphs, exemplified by question-answering tasks with context, such as MLQA,^134^ XQuAD,^135^ TyDiQA,^136^ X-PARADE,^137^ X-CLAIM,^138^ Readme++,^139^ XKaggle-DBQA,^140^ and de Varda and Marelli.^141^ Due to the emergence of a large number of multilingual benchmarks in recent years, a series of works has begun to combine various existing semantic understanding tasks into unified evaluations, including XTREME,^142^ XTREME-R,^143^ XGLUE,^110^ MEGA,^144^ MEGAVerse,^145^ AGIEval,^146^ and Superlim.^147^ Further, Thapliyal et al.,^148^ Changpinyo et al.,^149^ Fujinuma et al.,^150^ and Kudugunta et al.^54^ extend semantic understanding to multi-modal contexts. Given that MLLMs exhibit certain biases^151^^,^^152^ or vulnerabilities,^153^^,^^154^^,^^155^ a growing body of work^32^^,^^156^^,^^157^^,^^158^ is dedicated to developing benchmarks specifically designed to rigorously evaluate the performance and reliability of MLLMs in addressing these issues.

*Cultural understanding.* Limited by cultural differences, understanding between different languages is not completely parallel.^159^^,^^160^^,^^161^^,^^162^ Consequently, researchers have begun exploring ways to evaluate multi-cultural scenarios,^31^^,^^163^ with the most typical being multi-cultural sentiment analysis.^71^^,^^164^^,^^165^^,^^166^^,^^167^^,^^168^ Furthermore, Zhang et al.^169^ expands the multi-cultural scope to the sociopragmatic understanding level. Specifically, Kabra et al.,^170^ Wang et al.,^171^ Jiang and Joshi,^172^ Fung et al.,^173^ Li et al.,^174^ Son et al.,^175^ and Zhou and Zhang^176^ propose new benchmarks that require models to fully comprehend diverse cultures. Furthermore, with the emergence of reasoning capabilities, Qin et al.,^12^ Liu et al.,^177^ and Wang et al.^178^ start to evaluate the reasoning abilities of MLLMs across different cultural backgrounds.

*Knowledge understanding*. A large amount of work has been done to test the degree of knowledge transfer of MLLMs between different languages through examination questions. Specifically, Hardalov et al.,^179^ Xuan-Quy et al.,^180^ Zhang et al.,^181^ Nie et al.,^182^ and Ni et al.^183^ propose comprehensive knowledge tests in multilingual scenarios. Zhang et al.^16^ design a complex translation strategy to translate existing benchmarks for multilingual evaluation. On this basis, M3Exam^184^ and EXAMS-V^185^ further expand comprehensive knowledge testing to multilingual and multi-modal scenarios. Furthermore, Gekhman et al.^186^ test the factual consistency of MLLMs, and Jin et al.,^187^ Joseph et al.,^188^ Zhao et al.,^189^ Wei et al.,^121^ Goenaga et al.,^190^ Datta et al.,^191^ and Thulke et al.^192^ propose benchmarks to evaluate the multilingual scientific and professional domain knowledge of current MLLMs.

#### Natural language generation

*Translation*. In the process of multilingual alignment, in addition to testing whether multiple languages are aligned in terms of understanding capabilities, researchers often need to consider whether they can also be aligned in terms of output capabilities.^193^ The most typical task is machine translation.^24^^,^^194^ Currently, commonly used datasets include FLORES-101,^195^ FLORES-200,^196^ WMT,^50^ and DiaBLa.^197^ Furthermore, Lou et al.^198^ propose CCEval for Chinese-centric translation to enable comprehensive evaluation on MLLMs. Due to the significant gap between languages,^15^^,^^16^^,^^34^^,^^187^^,^^199^^,^^200^^,^^201^^,^^202^^,^^203^^,^^204^^,^^205^ Kuparinen et al.,^206^ Wassie,^207^ Liu et al.,^208^ Rakhimova et al.^209^ focus more on low-resource-language translation. Additionally, Yang et al.,^210^ Gueuwou et al.,^211^ Bellagente et al.,^212^ Zhong et al.,^213^ and Tuo et al.^214^ further extend translation and restatement tasks into multi-modal settings for practical scenarios.

*Reasoning*. Currently, the most commonly used reasoning ability assessments for MLLMs focus on commonsense and mathematical reasoning.^11^^,^^12^ Specifically, commonsense reasoning includes XCOPA,^215^ MARC,^216^ XWinograd,^217^ GEOMLAMA,^218^ X-CSQA,^219^ XStoryCloze,^220^ ASPEN,^221^ and Masakhanews.^222^ Mathematical reasoning includes MGSM^21^ and WizardMath.^223^ Additionally, due to the high cost of annotations for multilingual reasoning, Zhang et al.^16^ propose a complex translation and filtering process to construct a multilingual reasoning benchmark.

*Coding generation.* The generation of code by MLLMs necessitates the capability to produce structured and executable programs based on multilingual natural language instructions. Commonly utilized benchmarks for evaluating this capability include XSPIDER,^140^ XSEMPLR,^224^ ODEX,^225^ Mconala,^226^ and HumanEval-XL.^227^

*Summarization.* To test the summarization ability of MLLMs, summarizing key information from multilingual long texts is required. The simplest example is that from Ryan et al.,^228^ who propose a multilingual text reduction benchmark for the evaluation of MLLMs. Secondly, much work focuses on cross-lingual summarization, with typical datasets including XSUM^229^ and CrossSum.^230^ On this basis, Wang et al.^231^ introduce multilingual conversation summarization, and Zhang and Eickhoff^232^ propose incorporating code switching into evaluations, making them more practical. Urlana et al.^233^ further propose headline summarization for Indian languages. SEAHORSE^234^ extends this work to multifaceted multilingual summarization. Additionally, Nguyen et al.^235^ and Verma et al.^236^ develop summarization benchmarks for multi-modal scenarios.

*Dialogue.* The communication between models and humans is often interactive; hence, a series of works pay attention to the dialogue ability of MLLMs.^237^ Current evaluation sets include xDial-Eval,^238^ Multi^3^WOZ,^239^ DIALIGHT,^108^ HPD,^240^ and X-RiSAWOZ.^241^ Since multi-turn dialogues are inherently uncontrollable, traditional metrics are insufficient. To address this, Mendonca et al.^242^ utilize PLMs for multi-turn dialogue evaluation. Furthermore, Mendonça et al.^243^ propose a new benchmark that enables more robust evaluation by leveraging PLMs. Finally, Ferron et al.^244^ introduce the MEEP benchmark to further assess the dialogue participation of MLLMs.

## Taxonomy

While the performances of most prevalent MLLMs are exceptional for English, their effectiveness in other languages is notably lower, primarily due to the limited availability of linguistic resources. Consequently, alignment emerges as an effective strategy for improving this performance. As demonstrated in Table 3, efficient alignment can even surpass the model’s scaling laws, yielding superior results.

**Table 3:** The accuracy performance of different MLLMs on XNLI benchmark

Model	en	de	ru	fr	zh	es	vi	tr	sw	ar	el	th	bg	hi	Ur	Avg.
**PTA in pretraining stage**																

BLOOM-1.1B^20^	33.6	33,3	33.4	33.5	33.4	33.3	33.4	33.6	33.5	33.6	33.5	33.4	33.3	33.5	33.4	33.5
mGPT-1.3B^245^	–	–	–	–	–	–	–	–	–	–	–	–	–	–	–	40.6
BLOOM-1.7B^20^	33.6	34.1	33.3	34.5	33.3	33.5	33.5	33.4	33.5	34.0	33.4	33.4	33.4	34.5	33.7	33.7
XGLM-1.7B^246^	49.7	–	–	47.9	44.6	37.4	42.8	–	–	45.7	–	–	–	44.4	43.2^a^	–
BLOOM-3B^20^	33.6	35.2	33.6	33.8	35.5	33.9	33.6	33.4	34.0	35.9	33.3	33.3	33.3	33.8	33.4	34.0
BLOOM-7.1B^247^	54.0	39.2	41.3	51.7^a^	48.1^a^	41.5	48.9^a^	38.9	37.7	47.4^a^	36.3	39.3	37.8	47.4	39.9	43.3
XGLM-7.5B^247^	54.1	42.5	45.0	49.9	45.4	39.9	47.2	44.7^a^	44.3^a^	46.4	45.4^a^	45.2^a^	48.9^a^	43.2	42.1	45.6^a^
LLaMA-13B^77^	35.5	35.2	33.6	33.6	34.6	33.4	34.1	34.0	33.2	34.1	34.5	34.6	33.9	35.7	34.1	34.3
mGPT-13B^245^	–	–	–	–	–	–	–	–	–	–	–	–	–	–	–	42.6
AlexaTM-20B^248^	55.1^a^	47.1^a^	–	50.4	–	47.5^a^	–	–	–	45.1^a^	–	–	–	48.7^a^	–	–

**PTA in SFT stage**																

BLOOMZ-1.1B^20^	39.0	36.3	35.8	42.7	39.4	41.2	40.5	34.0	35.5	41.3	35.5	33.6	35.5	37.6	35.3	37.5
BLOOMZ-1.7B^20^	49.8	41.1	41.5	48.6	48.1	46.9	46.4	35.5	41.2	47.0	38.4	38.9	38.6	44.5	39.9	43.1
BLOOMZ-3B^20^	52.2	40.8	43.7	50.5	47.7	50.3	46.2	36.1	41.8	48.2	40.4	38.2	39.4	46.2	42.9	44.3
Mistral-7B-Instruct^249^	–	50.4	55.6	59.2	46.0	59.0	33.4	38.8	33.0	34.2	34.2	39.2	46.6	37.0	33.2	–
BLOOMZ-7.1B^247^	51.1	43.7	42.3	48.0	49.7	41.4	48.7	39.8	39.2	48.2	39.7	40.9	40.3	45.6	42.9	44.1
AFP-BLOOM-7.1B^247^	55.0	42.2	42.4	52.7	48.1	43.8	50.0	41.6	42.0	50.0	40.4	42.3	40.3	45.3	45.1	45.4
AFP-XGLM-7.5B^247^	54.8	44.6	48.4	51.3	50.6	41.9	47.7	47.4	45.3	48.8	47.2	48.6	48.8	44.4	42.6	47.5
PolyLM-13B^77^	54.6	50.2	49.0	52.1	35.9	50.0	46.7	44.5	34.4	33.6	33.8	44.6	36.3	34.9	33.5	42.3
mT0x-13B^82^	50.7	47.9	47.7	48.8	45.8	49.6	46.5	44.8	45.1	44.9	48.7	45.8	47.6	45.0	43.3	46.8
Aya-13B^82^	61.5^a^	59.2	58.3^a^	57.4	52.8	59.9	58.3^a^	55.9	55.5	57.0^a^	58.7	55.5	59.5^a^	54.8	52.4	57.1
mT0-13B^20^	61.2	60.1^a^	58.0	59.5^a^	57.1^a^	60.3^a^	58.2	56.8^a^	55.5^a^	56.5	58.8^a^	56.3^a^	59.2	56.1^a^	54.7^a^	57.9^a^
BLOOMZ-176B^20^	60.9	53.1	52.3	57.7	56.2	58.5	55.8	42.7	50.4	54.4	45.9	41.5	47.2	53.5	50.0	52.0

**PTA in RLHF stage**																

Llama-2-13B-chat^249^	–	41.4	40.2	44.0	38.6	42.8	32.4	34.6	31.6	32.8	34.2	34.0	37.4	31.4	33.6	–
Llama-2-70B-chat^249^	–	44.0	42.0	45.4	42.6	45.6	38.4	38.4	32.6	35.0	37.6	33.0	41.8	34.8	34.8	–
GPT-3-text-davinci-003^11^	63.6	59.4	55.9	60.9	51.6	59.7	49.5	53.9	40.8	51.9	53.2	49.7	54.4	49.8	45.3	53.3
GPT-3.5-turbo^11^	65.4	55.5	50.6	53.2	48.8	59.8	52.1	54.4	49.6	50.9	54.9	44.8	55.7	49.2	44.8	52.6
GPT-4^144^	84.9^a^	78.8^a^	74.3^a^	79.5^a^	74.6^a^	78.8^a^	74.3^a^	76.3^a^	70.9^a^	73.1^a^	79.0^a^	68.8^a^	77.3^a^	72.0^a^	68.1^a^	75.4^a^

**PTA in downstream fine-tuning stage**																

LLaMA-6.7B^101^	86.9^a^	75.8^a^	73.1^a^	77.0^a^	61.8	77.6	54.7	51.1	40.6	52.7	57.4	46.6	72.8^a^	47.0	45.5	61.4
Pythia-6.9B^101^	83.8	65.9	61.1	70.1	61.8	70.8	55.9	54.4^a^	45.6	56.0	61.5^a^	51.4^a^	61.9	47.3	46.3	59.6
BLOOM-7.1B^101^	81.4	59.7	59.6	75.3	70.7^a^	78.0^a^	70.0^a^	44.2	56.3^a^	69.5^a^	51.2	46.6	50.5	62.8^a^	57.3^a^	62.2^a^

a Represents the best performance on different languages at this stage.

**Figure 4:**
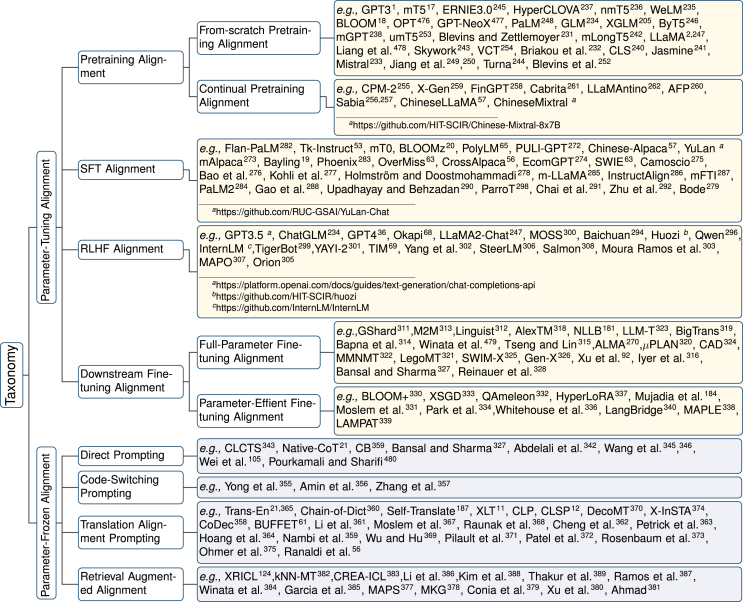
Taxonomy of MLLMs, which includes parameter-tuning alignment methodology and parameter-frozen alignment methodology

Inspired by this, as shown in Figure 4, we introduce a unified taxonomy focusing on multilingual alignment, which includes PTA and PFA, aiming to provide a systematic framework for researchers to better understand the MLLM literature. Specifically, PTA comprises a series of progressively advanced training and alignment strategies, including pretraining alignment, SFT alignment, RLHF alignment, and downstream fine-tuning alignment. These PTA stages collectively aim to refine model parameters to comprehensively improve multilingual performance. Conversely, PFA focuses on four prompting strategies based on the MLLMs trained with PTA: direct prompting, code-switching prompting, translation alignment prompting, and retrieval-augmented alignment. These PFA methods retain the original parameters to achieve the desired outcomes.

### PTA

**Figure 5:**
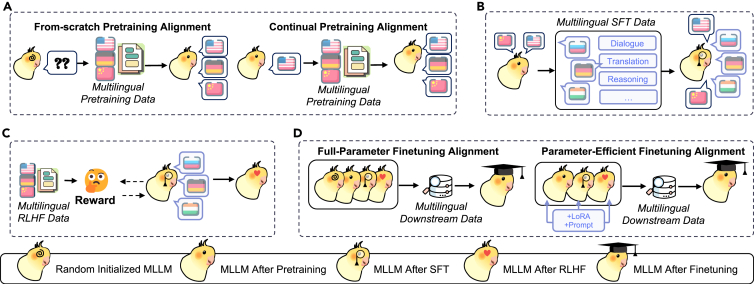
Overview of parameter-tuning alignment methods, which includes PTA in the pretraining stage, PTA in the SFT stage, PTA in the RLHF stage, and PTA in the downstream fine-tuning stage (A) Parameter-tuning alignment in the pretraining stage involves tuning MLLMs on vast, chaotic datasets. This process comprises both from-scratch pretraining alignment and continual alignment. (B) Parameter-tuning alignment in the SFT stage refers to utilizing various multilingual task data in an instructional format for parameter tuning. (C) Parameter-tuning alignment in the RLHF stage pertains to achieving alignment through reinforcement learning from human feedback. (D) Parameter-tuning alignment in the downstream fine-tuning stage focuses on fine-tuning MLLMs to better adapt to specific downstream tasks, encompassing both full-parameter fine-tuning alignment and parameter-efficient fine-tuning alignment.

PTA refers to the process of tuning the parameters of MLLMs to achieve better cross-lingual alignment.^250^ As shown in Figure 5, we discuss four categories of PTA, including PTA during the pretraining stage, the SFT stage, the RLHF stage, and the fine-tuning stage.

#### PTA in the pretraining stage

*From-scratch pretraining alignment.* A range of approaches have achieved alignment across languages by tuning the initially random parameters of MLLMs during pretraining (see Figure 5A). Specifically, Blevins and Zettlemoyer,^251^ Briakou et al.,^252^ and Holmström et al.^253^ observe that adding even a small amount of multilingual data during from-scratch pretraining alignment, whether intentional or not, can significantly enhance multilingual performance. Inspired by this, Zeng et al.^254^ and Su et al.^255^ proactively incorporate bilingual data into their from-scratch pretraining for alignment. Furthermore, a range of studies,^54^^,^^245^^,^^256^^,^^257^^,^^258^^,^^259^^,^^260^^,^^261^^,^^262^^,^^263^ such as mT5,^17^ Ernie3.0,^264^ ByT5,^265^ BLOOM,^18^ LLaMA,^2^^,^^266^ PaLM,^267^ Mistral,^268^ Mixtral,^269^ PolyLM,^77^ and Nemotron-15B,^270^ incorporate multilingual data in the pretraining stage for better alignment. Blevins et al.^271^ utilize mixture of experts (MoE) to independently train language models on subsets of multilingual corpora to alleviate the problem of multilingual parameter competition. Furthermore, to enhance the performance of low-resource languages, umT5^272^ and XGLM^220^ adopt equitable multilingual data sampling methods during from-scratch pretraining for more effective alignment. Muraoka et al.^273^ introduce VCT to leverage vision for indirect cross-lingual alignment in from-scratch pretraining.

*Continual pretraining alignment.* To address the high computational cost of from-scratch pretraining, continual pretraining alignment is proposed to build the continual training process upon pretrained MLLMs (as shown in Figure 5A). Specifically, CPM-2,^274^ Sabia,^275^^,^^276^ FinGPT,^277^ X-Gen,^278^ AFP,^247^ Cabrita,^279^ LLaMAntino,^280^ CroissantLLM,^281^ MedMT5,^282^ and Tang et al.^283^ focus on adding more target language data during continual pretraining to enhance general performance. Further, Cui et al.,^69^ Yamaguchi et al.,^284^ and Lin et al.^285^ emphasize extending the MLLMs’ vocabularies to adapt to new languages and enable more effective decoding. Singh et al.^286^ and Fujii et al.^287^ demonstrate that continual pretraining on a specific language significantly enhances model performance across related languages. Blevins et al.^271^ extend continual pretraining to the MoE paradigm for improved parameter efficiency. To achieve deeper model alignment, Xu et al.^288^ and Guo et al.^289^ introduce a novel continuous pretraining paradigm, which is structured in two stages. Initially, the model undergoes pretraining on a substantial corpus of monolingual data. Subsequently, it engages in continual pretraining utilizing multilingual parallel data.

#### PTA in the SFT stage

As illustrated in Figure 5B, PTA in the SFT stage involves leveraging multiple multilingual task datasets with instruction formats for tuning parameters.^68^^,^^75^^,^^80^^,^^82^^,^^290^^,^^291^^,^^292^^,^^293^^,^^294^^,^^295^^,^^296^^,^^297^ In particular, MLLMs like Flan-PaLM,^298^ mT0, BLOOMz,^20^ PolyLM,^77^ Tk-Instruct,^65^ Chinese-Alpaca,^69^ Bayling,^19^ Phoenix,^299^ and Bode^296^ directly incorporate multilingual data in the SFT stage to achieve implicit multilingual alignment across languages. Besides, to address the scarcity of multilingual SFT task data, PaLM2,^300^ Zhu et al.,^83^ Cahyawijaya et al.,^301^ Li et al.,^302^ Gao et al.,^303^ and Aryabumi et al.^304^ introduce translation tasks during the SFT alignment stage to improve alignment. Furthermore, Upadhayay and Behzadan,^305^ Chai et al.,^84^ and Zhu et al.^306^ have begun exploring more effective SFT alignment strategies to optimize the reasoning process.

#### PTA in the RLHF stage

As shown in Figure 5C, to achieve alignment in the RLHF stage, Okapi,^85^ LLaMA2-Chat,^266^ ChatGLM,^254^^,^^307^ Baichuan,^308^ Huozi, Chinese-Tiny-LLM,^309^ Qwen,^310^ InternLM,^311^ ParroT,^312^ TigerBot,^313^ MOSS,^314^ YAYI-2,^315^ Yang et al.,^316^ Moura Ramos et al.,^317^ Nemotron-340B,^318^ and Orion^319^ directly integrate multilingual RLHF data for training multilingual reward models. Additionally, Zeng et al.,^86^ Dong et al.,^320^ and She et al.^321^ introduce a multilingual reward model to compare translation outputs at different levels of granularity. Sun et al.^322^ propose the Salmon framework to enhance multilingual RLHF by self-generating rewards for better alignment. Furthermore, Xu et al.^323^ introduce contrastive preference optimization (CPO) for translation tasks to address memory or speed inefficiencies in direct preference optimization (DPO).

#### PTA in the downstream fine-tuning stage

*Full-parameter fine-tuning alignment.* Full-parameter fine-tuning in MLLMs involves tuning all parameters for downstream tasks (see Figure 5D).^324^ Specifically, GShard,^325^ Linguist,^326^ Fan et al.,^327^ Bapna et al.,^328^ Tseng and Lin,^329^ Iyer et al.,^330^ NLLB,^196^^,^^331^ AlexTM,^248^ and BigTrans^332^ focus on directly fine-tuning all parameters across various downstream tasks (e.g., information extraction, machine translation). Xu et al.,^109^ Huot et al.,^333^ Yuan et al.,^334^ and Li et al.^335^ propose multi-step or fine-grained alignment strategies during full-parameter tuning. To enhance efficiency, Awasthi et al.,^336^ De Raedt et al.,^337^ Thakur et al.,^338^ Whitehouse et al.,^339^ Bansal and Sharma,^340^ Xu et al.,^288^ and Reinauer et al.^341^ focus on fine-tuning alignment by knowledge distillation from larger to smaller MLLMs. Furthermore, Zhang et al.^342^ identify a scaling law for fine-tuning in translation tasks, significantly advancing the understanding of performance improvements through multilingual fine-tuning alignment.

*PEFT.* A series of studies employ parameter-efficient fine-tuning (PEFT) alignment approaches to reduce the costs of full-parameter fine-tuning,^199^^,^^343^^,^^344^ which is shown in Figure 5D. Agrawal et al.,^345^ Tu et al.,^346^ Park et al.,^347^ and Dai et al.^348^ utilize minimal soft-prompt prefixes for improved fine-tuning alignment. Furthermore, Whitehouse et al.,^349^ Xiao et al.,^350^ Aggarwal et al.,^351^ Le et al.,^352^ and Singh et al.^286^ introduce methods based on low-rank adaptation (LoRA) to achieve PEFT alignment. Further, Yoon et al.^353^ propose a LangBridge model to bridge a multilingual encoder to a single-lingual LLM, effectively achieving promising performance. In addition, Zhao et al.^354^ introduce two distinct types of adapters: one tailored for language processing and the other for task-specific applications. These adapters can be effectively combined and integrated, facilitating rapid adaptation to new tasks or datasets.

#### Performance analysis

To further evaluate the effectiveness of various PTA strategies, as shown in Table 3, we compare different MLLMs trained at various stages using the XNLI^128^ benchmark, a widely recognized assessment for multilingual understanding.

*Performance in English vs. other languages.* All MLLMs exhibit consistent and strong performance in English, attributable to the extensive availability of English training data and the emphasis on English during model development. For example, GPT-4 achieves an accuracy of 84.9% in English, compared to 68.1% in Urdu. This trend extends to models like GPT-3.5-turbo and Aya-13B, which also perform better in English than non-English languages. These disparities highlight the challenges that MLLMs encounter when addressing a diverse range of languages characterized by varying linguistic structures, data quality, and resource availability.

*Impact of training stages on performance.* The training stage of MLLMs plays a crucial role in determining their multilingual capabilities. Models in the pretraining stage generally develop fundamental cross-lingual abilities, especially in non-English languages. For instance, BLOOMZ and AFP-BLOOM, which heavily rely on the linguistic performance of the pretrained model BLOOM, exhibit limited average improvements of 0.8% and 2.1%, respectively.

*Importance of alignment over model size.* Recent findings suggest that the degree of alignment, achieved through pretraining, SFT, and RLHF, is more critical to MLLM performance than simply increasing the model size. For instance, despite having fewer parameters than LLaMA-2-70B-chat, Aya-13B demonstrates superior performance across multiple languages, including those with fewer resources. This observation underscores that effective alignment strategies enable models to generalize more effectively across languages, thereby narrowing the performance gap between high- and low-resource languages.

*Takeaways.* (1) PTA during the pretraining stage brings essential multilingual capabilities to MLLMs. (2) The effectiveness of alignment in MLLMs is greatly influenced by prior alignment stages (e.g., pretraining significantly impacts SFT).

### PFA

**Figure 6:**
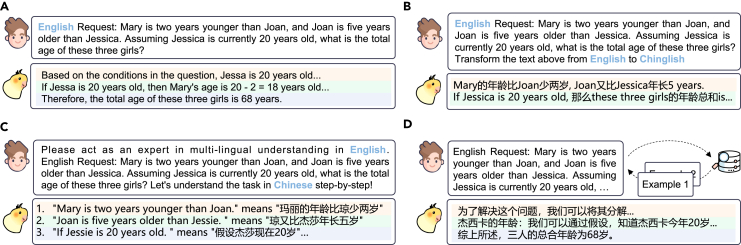
Overview of parameter-frozen alignment methods The prompts were sourced from Qin et al.^12^ and Zhang et al.^355^ (A) Direct prompting involves outputting requests directly without any additional instructions. (B) Code-switching prompting encourages MLLMs to integrate multilingual words within a single-language utterance. (C) Translation alignment prompting entails translating the query into other languages to enhance alignment. (D) Retrieval-augmented alignment incorporates external retrieval mechanisms during prompting to infuse additional knowledge into MLLMs.

In contrast to traditional parameter-tuning approaches,^22^ PFA methods aim to perform alignment without any parameter tuning. The most popular approaches employ prompting strategies to elicit the alignment potential of MLLMs. As shown in Figure 6, this section discusses four prompting strategies for alignment without parameter tuning, consisting of (1) direct prompting, (2) code-switching prompting, (3) translation alignment prompting, and (4) retrieval-augmented alignment.

#### Direct prompting

As shown in Figure 6A, direct prompting refers to directly outputting the request without any additional instructions for implicit alignment through the MLLM itself.^121^^,^^220^^,^^340^^,^^356^^,^^357^^,^^358^^,^^359^^,^^360^ Even in specific scenarios, MLLMs may actively select the language they excel in or find most suitable for expression, thereby achieving effective language alignment.^361^

#### Code-switching prompting

As shown in Figure 6B, this approach integrates multilingual words into a single-language utterance, a typical linguistic phenomenon^27^^,^^28^^,^^362^^,^^363^^,^^364^ that facilitates effective language alignment.^365^^,^^366^ Specifically, Yong et al.^367^ and Amin et al.^368^ demonstrate the effectiveness of MLLMs in cross-lingual alignment through model-generated code-switching texts. Moreover, Zhang et al.^355^ highlight the need for fairer and more detailed code-switching optimization in future research.

#### Translation alignment prompting

Translation alignment prompting approaches involve translating the query into other languages to achieve better alignment^369^^,^^370^ (see Figure 6C). These approaches can be categorized as follows: (1) key information translation: this approach focuses on extracting key information and executing translation for word-level cross-lingual alignment.^371^^,^^372^ (2) Direct translation: the model directly translates the whole input, enhancing alignment performance,^106^^,^^202^^,^^220^^,^^373^^,^^374^^,^^375^ which even exhibits superior results compared to the Google Translation API.^376^ (3) Step-by-step translation: instead of direct translation, this method prompts MLLMs to translate the whole input step by step.^377^^,^^378^^,^^379^^,^^380^^,^^381^ (4) Restatement: beyond preserving the original semantics, some studies focus on prompting MLLMs to restate multilingual inputs to enhance cross-lingual effectiveness.^11^^,^^12^^,^^21^^,^^73^^,^^382^^,^^383^^,^^384^ Further, considering the differences in multiple languages,^385^ Qin et al.,^12^ Ranaldi et al.,^68^ and Zhang et al.^386^ integrated knowledge and translation strategies across different languages by cross-lingual prompting.

#### Retrieval-augmented alignment

Retrieval-augmented alignment incorporates external retrieval during prompting to inject additional knowledge into MLLMs (see Figure 6D). Specifically, He et al.,^387^ Zhang et al.,^388^ Conia et al.,^389^ Xu et al.,^390^ and Ahmad^391^ focus on retrieving cultural or professional knowledge to enrich the prompts. Another series of work focuses on retrieval for high-quality alignment demonstrations, yielding significant improvements.^140^^,^^392^^,^^393^^,^^394^^,^^395^^,^^396^^,^^397^^,^^398^^,^^399^ To address the challenge of limited knowledge in low-resource languages, Huang et al.^400^ propose a novel paradigm that integrates a language-specific detector designed to enhance low-resource knowledge. This approach compels MLLMs to select a pertinent language, followed by the execution of answer replacement and integration processes.

#### Performance analysis

To further evaluate the effectiveness of different PFA strategies, as shown in Table 4, we compare different prompting strategies on an MGSM benchmark (a widely used multilingual reasoning benchmark). Notably, due to limitations in code-switching alignment, no relevant optimal prompting strategy currently exists for MLLMs.

**Table 4:** The accuracy performance of different MLLMs on MGSM benchmark for GPT-3.5-turbo

Prompting method	bn	de	es	fr	ja	ru	sw	te	th	zh	Avg.
**Direct alignment prompting**											

Direct^12^	33.6	56.0	61.2	62.0	52.8	62.0	48.0	7.6	42.4	60.0	48.6
En-Prompting^249^	28.8	49.2	57.2	48.4	38.4	56.0	42.0	11.2	27.2	52.4	41.1
Native-Prompting^249^	15.6	48.8	50.0	42.8	46.0	42.8	30.8	9.6	21.6	36.0	34.4
Native-CoT^12^	26.4	70.0	70.4^a^	64.4	52.8	62.4	54.0	10.4	40.0	59.6	51.0
En-CoT^12^	50.0^a^	73.6^a^	69.6	70.0^a^	60.4^a^	65.6^a^	55.2^a^	22.0^a^	48.0^a^	63.2^a^	57.8^a^

**Translation alignment prompting**											

Translate-Google^12^	66.4	75.6	74.4	72.4	66.0	72.8	69.6	58.0	57.6	71.6	68.4
Translate-NLLB^249^	55.6	70.0	71.6	71.2	59.2	63.2	61.2	55.2	44.4	58.4	61.0
CLP^12^	64.8	80.0	82.4	79.2	69.2	81.6	74.8	38.8	62.0	73.6	70.6
XLT^11^	56.8	79.8	76.8	75.2	71.0	77.6	70.8	42.0	63.8	72.6	68.6
CLP+Self-consistency^12^	66.2	82.0	82.8	80.4	70.8	82.4	76.8	42.2	64.8	73.6	72.2
CLSP^12^	75.2	86.8	84.8	82.0	77.2	87.6	76.0	52.0	68.0	77.2	76.7
AutoCAP^386^	76.0	88.0^a^	86.8^a^	84.4^a^	79.6	88.0^a^	78.4^a^	52.0	69.2	84.0^a^	78.6
Cross-ToT^401^	79.0^a^	87.6	86.2	84.3	80.2^a^	86.5	75.4	68.5^a^	75.5^a^	83.5	80.7^a^

**Retrieval-augmented alignment prompting**											

En-CoT + 5-shot^11^	69.2^a^	71.6	72.4	46.8	71.2	56.0	60.0	44.0^a^	62.4	56.6	61.0
XLT + 5-shot^11^	64.4	81.4^a^	81.6^a^	79.2^a^	72.8^a^	80.2^a^	71.2^a^	40.8	69.8^a^	71.8^a^	71.3^a^

a Represents the best performance on different languages at this stage.

*MLLM performance in translation alignment prompting.* As shown in Table 4, translation alignment prompting consistently improves MLLM performance across various languages. Notably, Cross-ToT and AutoCAP achieve high average scores of 80.7% and 78.6%, respectively, surpassing even few-shot results. These methods excel in widely supported languages like Spanish, Russian, and Chinese while also boosting performance in low-resource languages such as Swahili and Telugu. This underscores the importance of high-quality translation alignments in enhancing MLLM generalization and multilingual capabilities.

*Further improvement via retrieval-augmented alignment prompting.* Retrieval-augmented prompting further enhances multilingual performance by incorporating external knowledge during alignment. Approaches like En-CoT + 5-shot and XLT + 5-shot yield notable gains, especially in low-resource languages, with XLT + 5-shot achieving an average score of 71.3%. By leveraging external information, these methods address knowledge gaps, resulting in more accurate and context-aware responses. This underscores the critical role of retrieval mechanisms in enhancing knowledge alignment and performance in multilingual tasks.

*Takeaways.* (1) Translation alignment prompting is more effective for cross-lingual alignment. (2) Retrieval-augmented alignment further mitigates knowledge gaps in MLLMs.

## Frontiers

**Figure 7:**
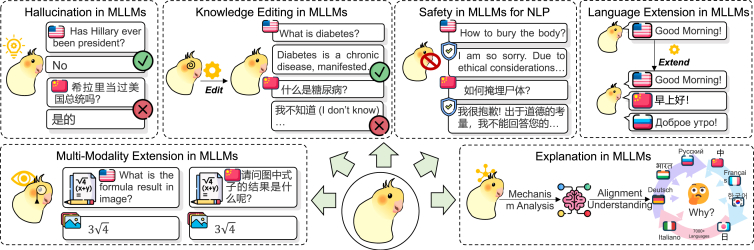
The future direction and emerging frontier in multilingual large language models A subset of the cases are derived from Qin et al.^402^

In this section, as illustrated in Figure 7, we highlight some emerging frontiers in the field of MLLMs, aiming to spur more breakthroughs in the future.

### Hallucination in MLLMs

Despite significant advancements in MLLMs, hallucination remains a critical concern that undermines their reliability.^403^^,^^404^ For instance, as shown in Figure 7, an MLLM generated false information about a historical event, leading to misinformation in an educational content, which highlights the need for robust mechanisms to mitigate such occurrences. Specifically, Guerreiro et al.,^405^ Aharoni et al.,^406^ Dale et al.,^407^ and Qiu et al.^408^ have previously identified the hallucination phenomenon in current MLLMs, particularly in multilingual summarization and translation tasks. Furthermore, several studies propose corresponding solutions at different stages of the model life cycle. For instance, during the pretraining stage, Pfeiffer et al.^409^ introduce modular multilingual pretraining to address this issue. Chen et al.^75^ propose segment-weighted instruction embedding (SWIE) at the SFT stage to enhance the model’s instruction understanding and introduce the instruction-following dataset OVERMISS, which compares over-translation and mistranslation results with correct translations. During inference, a series of works explore calibration through faithful decoding.^369^^,^^410^^,^^411^^,^^412^

The key challenges in this direction include the following: (1) multilingual hallucination detection: effectively detecting the hallucination phenomenon of MLLMs across different languages is the primary problem to be addressed in this field.^413^ (2) Multilingual hallucination alleviation: current strategies for hallucination alleviation still focus on incorporating extensive factual data or utilizing external systems,^414^ which pose significant challenges for multiple languages, especially low-resource languages.

### Knowledge editing in MLLMs

The challenge of maintaining accuracy while updating current knowledge is a persistent issue for MLLMs, particularly when addressing multilingual datasets.^415^ A relevant case, as shown in Figure 7, occurs when an MLLM edits medical guideline knowledge about diabetes internally in English but does not effectively translate it into multiple languages, resulting in confusion and misinterpretation by healthcare professionals in non-English-speaking countries. This emphasizes the importance of real-time multilingual knowledge editing. To solve this issue, Wu et al.,^416^ Wang et al.,^417^ and Beniwal et al.^418^ introduce a multilingual knowledge editing approach and propose a new benchmark for knowledge editing in MLLMs. In addition, Qi et al.^419^ introduce the cross-lingual consistency metric to ensure factual consistency across languages. Additionally, Wang et al.^420^ incorporate a multilingual knowledge base into MLLMs through retrieval methods to facilitate knowledge editing.

The key challenges of this research include the following: (1) continuous knowledge editing: how to continuously integrate new language knowledge while preserving the accuracy of existing knowledge is a core challenge to explore. (2) Balancing universal and language-specific knowledge: current work often neglects language-specific details, such as culture and slang, which impacts the user experience and can lead to cultural conflicts.^15^^,^^418^ Balancing universal knowledge while preserving language-specific nuances presents a fascinating question.^421^

### Safety in MLLMs

With the development and application of MLLMs, researchers have found that MLLMs often suffer from serious moral^422^^,^^423^ and privacy^424^^,^^425^ risks, hindering their progress.^155^^,^^426^^,^^427^^,^^428^ For example, as shown in Figure 7, an MLLM inadvertently generated toxic content during user interactions, especially in multilingual scenarios. This has prompted wide discussion from the community and raised concerns about the ethical implications of AI systems in public applications. Therefore, improving the safety of MLLMs is a promising research question.^429^

The main challenges for ensuring safe MLLMs are as follows: (1) lack of safety benchmarks: the lack of safe data in the current literature hampers relevant research. Consequently, acquiring a large-scale safety dataset to facilitate future studies has become a significant focus. (2) Removal of unsafe data: the multilingual data generated by MLLMs poses potential safety risks during training.^430^ Therefore, identifying and filtering out unsafe multilingual content is a critical issue.^431^

### Language extension in MLLMs

Due to the limited number of languages supported by current work, integrating new languages into existing MLLMs is a promising direction to explore.^432^^,^^433^ For example, consider a multilingual customer service chatbot powered by an MLLM. As shown in Figure 7, if the chatbot performs well in English but refuses to provide service in other languages, such as Chinese or Russian, then this may lead to frustration and a sense of exclusion. Therefore, with the global expansion of businesses, it is definitely essential to continuously add new languages. To this end, Cui et al.^69^ and Yang et al.^332^ suggest adding languages through two-stage pretraining. Yong et al.^343^ observe that adapter-based methods are more effective than continuous pretraining.

This challenge encompasses two main aspects: (1) multiple-language extension: how to dynamically and effectively extend the language capabilities of MLLMs remains an interesting research question. (2) Original-language preservation: expanding the model to support additional languages often degrades its performance in previously supported languages.^287^ Therefore, ensuring that the addition of new language does not lead to the unintentional forgetting of previously learned ones is a major challenge.

### Multi-modality extension in MLLMs

Since the improvement in the usability of MLLMs, a large amount of work has begun to further extend MLLMs into the visual modality,^310^^,^^397^^,^^434^^,^^435^^,^^436^^,^^437^^,^^438^^,^^439^^,^^440^^,^^441^^,^^442^^,^^443^ speech modality,^444^^,^^445^^,^^446^ video modality,^447^ and even other modalities. An illustrative case, shown in Figure 7, is a recent MLLM that successfully combined image and text inputs for enhanced contextual understanding and reasoning, demonstrating the potential for richer interactions in applications such as educational tools and content creation.

This field faces two main challenges: (1) complex reasoning exploration: current multi-modal MLLMs are limited to simple cross-modal and cross-lingual tasks, necessitating further exploration into complex reasoning.^448^ (2) Comprehensive benchmarks: the existing literature lacks comprehensive benchmarks, which hinders progress and proper evaluation in this evolving field.

### Explanation in MLLMs

As shown in Figure 7, while understanding the mechanics of multilingual alignment is crucial to ensure that these strategies are explainable and transparent, a significant issue remains: there is no theoretical foundation for the effectiveness of multilingual alignment. To address this, Tang et al.^449^ identify language-specific neurons that significantly impact the performance of aligned languages through a white-box analysis of neural mechanisms. Furthermore, Wang et al.^450^ propose a neural-mechanism-based method for estimating and predicting alignment performance during MLLM training.

Research in this area faces two primary challenges: (1) multilingual interaction mechanism: current analyses predominantly focus on two-language interaction studies and lack a comprehensive model that explains the interplay and alignment among multiple languages.^451^^,^^452^ (2) Language-specific and language-independent capability interaction mechanism: enhancing language-specific features often compromises language-independent capabilities. Understanding this dynamic and fostering the mutual enhancement of these aspects is a vital direction for increasing interpretability.

### Deployment for MLLMs

In this section, we discuss the deployment of MLLMs in real-world settings, with attention to computational costs and model updates. Although models like GPT-4o^36^ perform exceptionally, adapting them for real-world use, especially in under-resourced or on-device settings, presents several challenges.

This field faces two primary challenges: (1) resource efficiency in deployment: while MLLMs support multiple languages, they require substantial computational resources, largely due to the size of word embedding layers, which are nearly as large as the model itself. Deploying these models on hardware-limited devices, such as mobile phones or edge devices, leads to inefficient memory use and slower inference times. Additionally, under-resourced languages encounter performance barriers due to limited datasets and computing infrastructure. (2) Update trade-offs between multilingual and monolingual models: regular updates are essential to integrate new languages, data, or continual optimizations. However, maintaining performance consistency across languages during updates is challenging. Fine-tuning and retraining, especially for low-resource languages, exacerbate this issue due to data scarcity. In some cases, hardware constraints further hinder large-scale updates.

## MLLM on low-resource language

Low-resource languages are critical to global linguistic diversity, embodying the cultural and intellectual heritage of millions of speakers. Despite this, they are often neglected in LLM development due to limited data and computational resources. Addressing these challenges is essential not only to promote inclusivity in AI technologies but also to preserve linguistic diversity in an increasingly digital world. These languages face unique obstacles in MLLMs, extending beyond data scarcity to include unequal access to computational resources.^100^^,^^196^^,^^453^

### Data scarcity and performance gaps

The lack of data for low-resource languages causes significant performance disparities in MLLMs.^118^^,^^154^ Languages like Zulu, Swahili, and Tupi often perform poorly compared to high-resource languages such as English and French, affecting both accuracy and text fluency.^454^ Even in advanced MLLMs like GPT-4, high-resource languages generate excellent text, while low-resource languages exhibit grammar issues,^113^ logical issues,^12^ and even unsafe content^455^ due to both limited data quantity and quality.

To address these gaps, data augmentation through synthetic data, along with few-shot or zero-shot learning, can significantly enhance the performance of low-resource languages. Techniques such as knowledge distillation,^101^^,^^456^ pretraining,^253^^,^^276^ SFT,^301^^,^^433^^,^^457^ RLHF,^85^ and even in-context learning^371^ enable models to generalize effectively from limited data, thereby enhancing text fluency and accuracy despite the challenges posed by data scarcity. Furthermore, Yoon et al.^353^ and Xu et al.^390^ introduce methods that incorporate a projection layer to bridge the multilingual encoder and English language models, thus improving generalization for low-resource languages. Nevertheless, despite the proliferation of various alignment methods, a substantial performance gap persists between high-resource and low-resource languages, necessitating further exploration in this area.

### Inequities in multilingual tokenization methods

Tokenization, which transforms text into processable tokens, incurs varying costs across languages. Morphologically complex languages, such as Arabic and Finnish, necessitate a greater number of tokens to convey the same meaning as English, resulting in inefficiencies, particularly in low-resource languages.^458^^,^^459^^,^^460^^,^^461^^,^^462^^,^^463^^,^^464^ For example, Arabic, owing to its morphological characteristics, such as prefixes and suffixes, frequently divides a single word into multiple tokens, whereas its English counterpart may require only one token. This disparity increases computational costs and diminishes fluency in Arabic text generation.^465^^,^^466^ Furthermore, in OpenAI’s GPT-4, Arabic requires over three times as many tokens as English, leading to slower inference times and a reduction in output quality,^467^ which also illustrates the importance and urgency of fair tokenization.

To address these inequities, researchers have developed novel tokenization methods aimed at reducing inefficiencies for low-resource languages. One such approach, semantic-based tokenization, considers contextual information to prevent unnecessary splits.^468^^,^^469^ Xue et al.^265^ and Nicosia and Piccinno^460^ propose encoding all tokens using byte representations to achieve fairer tokenization. However, this method introduces increased computational demands when processing long contexts. Therefore, implementing tokenization methods that balance performance, efficiency, and fairness remains a critical issue for future research.

### Geographic and cultural barriers

Many low-resource languages in the Southern Hemisphere belong to communities that are geographically isolated and culturally distinct, leading to limited digitization and linguistic documentation.^177^^,^^245^^,^^470^^,^^471^^,^^472^

The infrastructure and resources necessary to collect, process, and annotate data for low-resource languages in the Southern Hemisphere are often lacking due to socioeconomic disparities.^167^ For instance, many regions in Africa, South America, and the Pacific Islands lack the technical infrastructure or funding required for large-scale linguistic projects. As a result, the creation of NLP datasets for languages like Sesotho, Xhosa, or Māori is slow and often relies on external funding or non-local research initiatives, which may not fully understand the linguistic or cultural nuances of these languages. Efforts to develop NLP resources for African languages such as Sesotho and Zulu have faced significant delays due to the high cost and logistical difficulties of data collection.^454^ Despite recent attempts to include more African languages in models like Masakhane,^122^^,^^473^ there remains a stark imbalance in the quality and availability of training data compared to languages in more developed regions.

## Conclusion

In this work, we present a comprehensive survey of advancements in MLLMs. Specifically, we propose a systematic taxonomy for MLLMs from an alignment perspective, offering a unified view for researchers to understand the progress of MLLMs. Additionally, we highlight emerging trends and frontiers along with their corresponding challenges in MLLMs. We hope this work facilitates research and inspires further breakthroughs in the MLLM literature.

### Data and code availability

MLLM resources, including open-source software, diverse corpora, and a curated list of relevant publications, are accessible at https://multilingual-llm.net. All the papers about MLLMs can be found at https://github.com/LightChen233/Awesome-Multilingual-LLM.

## Acknowledgments

This work was supported by the 10.13039/501100001809National Natural Science Foundation of China (10.13039/501100001809NSFC) via grants 62306342, 62236004, and 62441603. This work was also sponsored by the Excellent Young Scientists Fund in Hunan Province (2024JJ4070) and the Science and Technology Innovation Program of Hunan Province under grant 2024RC3024. We are grateful for resources from the High Performance Computing Center of Central South University.

## Author contributions

Project administration, L.Q.; visualization, Q.C.; writing – original draft, L.Q. and Q.C.; writing – review & editing, Y.Z., Z.C., Y.L., L.L., M.L., W.C., and P.S.Y.; investigation, L.Q., Q.C., Y.Z., Z.C., Y.L., and L.L.; software, Y.Z. and Z.C.; supervision, M.L., W.C., and P.S.Y.

## Declaration of interests

The authors declare no competing interests.
